# Osteopontin is a prognostic circulating biomarker in patients with neuroendocrine neoplasms

**DOI:** 10.1007/s00432-023-04979-6

**Published:** 2023-06-15

**Authors:** Evelyn Kidess, Yvonne Giesecke, Ines Eichhorn, Raphael Mohr, Henning Jann, Christian Fischer, Bertram Wiedenmann, Christoph Roderburg, Frank Tacke, Michael Sigal

**Affiliations:** 1 Department of Hepatology and Gastroenterology, Campus Virchow Clinic and Campus Charité Mitte, Charité University Medicine, Berlin, Germany; 2 Clinic for Gastroenterology, Hepatology and Infectious Diseases, University Hospital Düsseldorf, Medical Faculty of Heinrich Heine University Düsseldorf, Düsseldorf, Germany

**Keywords:** Osteopontin, Biomarker, Neuroendocrine neoplasm, Chromogranin A, Survival

## Abstract

**Purpose:**

Osteopontin (OPN), also called secreted phosphoprotein 1 (SPP1) is a matricellular glycoprotein whose expression is elevated in various types of cancer and which has been shown to be involved in tumorigenesis and metastasis in many malignancies. Its role in neuroendocrine neoplasms (NEN) remains to be established. The aim of the study was to analyze plasma concentrations of OPN in patients with NEN and to explore its diagnostic and prognostic value as a clinical biomarker.

**Methods:**

OPN plasma concentrations were measured in a total of 38 patients with histologically proven NEN at three different time points during the course of disease and therapy (at the start of the study, after 3 and 12 months, respectively) as well as in healthy controls. Clinical and imaging data as well as concentrations of Chromogranin A (CgA) and Neuron Specific Enolase (NSE) were assessed.

**Results:**

OPN levels were significantly higher in patients with NEN compared to healthy controls. High-grade tumors (grade 3) showed the highest OPN levels. OPN levels were neither different between male and female patients nor between different primary tumor sites. OPN correlated significantly with corresponding NSE levels, while there was no correlation with Chromogranin A. High OPN levels above a cutoff value of 200 ng/ml at initial analysis predicted a worsened prognosis with significantly shorter progression-free survival of patients with NEN, which also held true within the subgroup of well-differentiated G1/G2 tumors.

**Conclusion:**

Our data indicate that high baseline OPN levels in patients with NEN are predictive of an adverse outcome with shorter progression-free survival, even within the group of well differentiated G1/G2 tumors. Therefore, OPN may be used as a surrogate prognostic biomarker in patients with NEN.

## Introduction

Neuroendocrine neoplasms (NENs) are a group of rare, very heterogeneous cancers (Kunz 2015). Nevertheless, the incidence and prevalence of NENs has been rising globally: it is estimated, that every year over 12,000 people in the United States are diagnosed with NET (Dasari et al. 2017; Oberg et al. 2013). The prognosis of NENs is largely dependent on the histopathological assessment, which is based on the World Health Organization (WHO) classification of 2019 (Rindi et al. 2022). Disease stage and tumor grading are important factors of the classification and largely correlate with the prognosis (Baur et al. 2016). Whereas well-differentiated NEN have mostly favorable prognosis, poorly differentiated NEN (neuroendocrine carcinoma, NEC) are highly proliferative (Ki67 index > 20%) with a median overall survival of 11–17 months (Rinke and Gress 2017; Rindi et al. 2022).

When diagnosed at an early stage, surgical resection can be performed, leading to significantly improved overall survival. Due to their often-indolent growth, NENs are frequently diagnosed at a late stage, when metastases have occurred (Modlin et al. 2008).

The tumor marker Chromogranin A (CgA) is commonly used in clinical practice for monitoring patients with NEN (Lindholm and Oberg 2011). Serum concentration of Neuron-Specific Enolase (NSE) can also be found elevated in up to 45% of patients with NEN and seems to correlate with a worsened prognosis, also in cases of normal levels of CgA (Appetecchia et al. 2018; Kulke et al. 2019). CgA is a glycoprotein, which is expressed in large core vesicles of neuroendocrine cells (Borges et al. 2010). Increased levels of CgA have been associated with Neuroendocrine Neoplasms (NENs) from many different sites, including the gastroenteropancreatic tract (Diez et al. 2013), the bronchopulmonary system (Caplin et al. 2015; Pericleous et al. 2018), as well as pheochromocytomas, paragangliomas, medullary thyroid carcinoma, as part of multiple endocrine neoplasia type 1 (MEN-1), Von-Hippel Lindau (VHL) syndrome (Cives and Strosberg 2018; Bottoni et al. 2015), and others.

Currently, even after curative surgery, surveillance is recommended at certain intervals using imaging and CgA measurement (Knigge et al. 2017). While changes of CgA levels in individual patients can be useful as surrogate for tumor progression, the levels do not reflect the aggressiveness of the tumor and further most aggressive G3 tumors often express less CgA compared to well differentiated tumors (Campana et al. 2007; Marotta et al. 2012).

NSE, an enzyme which is specific for neurons and neuroendocrine cells (Isgro et al. 2015), has emerged as another biomarker, which is frequently increased in high-grade G3 tumors (van Adrichem et al. 2016), although its clinical value is under debate due to limited sensitivity (Pavel et al. 2020; Modlin et al. 2008; Rindi et al. 2022).

Osteopontin (OPN) is a non-collagenous bone matrix protein, produced by osteocytes, osteoblasts and hematopoietic cells (Wang and Denhardt 2008; Kita et al. 2006). Apart from promoting physiological responses, e.g. regulation of bone mineralization, promoting cell adhesion and migration, as well as recruitment of macrophages, the importance of OPN in cancer progression is becoming increasingly acknowledged (Zhao et al. 2018; Hao et al. 2017). The role of OPN in malignancies has been demonstrated in several different cancer types, including breast, prostate and colorectal cancer, melanoma, osteosarcoma and glioblastoma (Zhao et al. 2018; Amilca-Seba et al. 2021). To date there is no study, which assessed the implication of OPN for NENs. Therefore, we sought to evaluate the role of OPN in this cancer entity, and evaluate its utility as a prognostic biomarker.

## Methods

### Patient recruitment and study cohort

From December 2013 to October 2016, 38 patients diagnosed with Neuroendocrine Neoplasms were enrolled in our study at our tertiary center for Neuroendocrine Tumors at the Charité University Hospital. Prior to sample collection, patients’ informed written consent was obtained. The local ethics committee approved the study (EA EA1/229/17). From December 2013 to March 2018 blood samples from those thirty-eight patients were collected at three time points during the course of disease and therapy (i.e., at the beginning of the study / beginning of therapy, after three months, and after 12 months, respectively).

The levels of circulating OPN were evaluated as a potential biomarker for NETs. In all patients, prior histopathological analyses of tumor tissue obtained by tumor resection or biopsy proved the presence of a NEN. Tumor grading was performed in accordance with the WHO guidelines.

We also collected blood samples from ten healthy controls with no history of malignancies, which served as control samples.

### Sample procession and measurement of OPN levels

After the collection of patients’ and healthy donors’ blood samples, they were subjected to a centrifugation step for 10 min at 3000 g, and plasma aliquots of 1 mL were frozen at − 80 °C until further analyses. In total, we analyzed plasma from 114 patient samples and 10 healthy donors.

Plasma levels of OPN were measured by using an enzyme-linked immunosorbent assay (ELISA) according to the manufacturer´s instructions (No. 27158, Immuno-Biological Laboratories (IBL) International GmbH Flughafenstrasse 52a 22,335 Hamburg, Germany). This kit uses two types of highly specific antibodies. Tetra Methyl Benzidine (TMB) is used as coloring agent (Chromogen). The epitopes of the used antibodies are as follows: (1) Coating Antibody: Anti-Human OPN (O-17) Rabbit IgG Affinity Purify. (2) Labeled Antibody: Anti-Human OPN (10A16) Mouse IgG MoAb Fab'-HRP. The measurement range amounts from 5 to 320 ng/mL. The sensitivity of the reaction is 3.33 ng/mL.

All samples were measured in duplicates.

In parallel, blood samples for analyses of CgA and NSE levels were measured as a part of the standard workflow for NEN patients at Labor Berlin, the central laboratory of Charité University Hospital Berlin, Germany. The CgA and NSE analyses each were performed with the same method at all time points. Also, the normal upper limits remained the same (CgA < 102 ng/ml; NSE < 16.3 ug/L).

### Statistical analyses

All assays were performed in replicates. Results are displayed as violin plots or box plots. Parametric data were compared using student’s *t*-test, nonparametric data were compared using the Mann–Whitney *U*-test or the Kruskal–Wallis test for multiple group comparisons.

A *p*-value of < 0.05 was considered statistically significant. Statistical analyses were performed by using GraphPad Prism software.

## Results

### Patient characteristics

In the current study, plasma from 38 patients (22 female and 16 male, respectively), were collected at three different time points during the course of the disease for analysis of OPN levels. The standard biomarker for NENs, CgA, but also NSE, were analyzed in parallel. Further, blood from ten healthy donors for analyses of OPN levels served as controls. Within the control group, 5 were female and 5 were male participants. The median age was 40 years (29–58 years).

At the time point of inclusion into the study, the median age of the patients was 63 years (28–85). In the majority of cases, the primary tumor was localized in the ileum (*n* = 20) or the pancreas (*n* = 13). Further primary tumor locations included the kidney (*n* = 1), mammary gland (*n* = 1), lung (*n* = 1) and carcinoma of unknown primary (*n* = 2). The median Ki-67 index was 8% (range 1–40%). The majority of patients showed a G2 tumor (58%) and no functionality (68%). In most cases, the tumor had metastasized to lymph nodes and the liver at the time of inclusion into our analyses.

Of 38 patients, 26 showed elevated levels of Chromogranin A (68%), and 27 showed elevated levels of NSE (70%). Patient characteristics are summarized in Table 1.

**Table 1:** Patient characteristics (total number of patients *n* = 38)

Age at diagnosis, median (years)	63 (28–85)
Gender	
Male	16 (42%)
Female	22 (58%)
Primary tumor localization	
Ileum	20 (53%)
Pancreas	13 (34%)
Other	5 (13%)
Ki-67 (%)	
≤ 2	14 (37%)
3–20	22 (58%)
> 20	2 (5%)
Grading	
G1	14 (37%)
G2	22 (58%)
G3	2 (5%)
Functional disease	
Yes	12 (32%)
No	26 (68%)
Somatostatin Receptor Status	
Positive	24 (63%)
Negative	4 (11%)
Not evaluated	10 (26%)
Metastases	
Liver	27 (71%)
Lymph nodes	20 (53%)
Bone	4 (11%)
Lung	2 (5%)
Kidney	1 (3%)
None	5 (13%)
Chromogranin A (ng/ml) at first blood draw	
< 102	12 (32%)
> 102	26 (68%)
Neuron Specific Enolase (μg/l) at first blood draw	
< 16.3	11 (30%)
> 16.3	27 (70%)

### Osteopontin (OPN) levels are elevated in patients with neuroendocrine malignancies

**Fig. 1:**
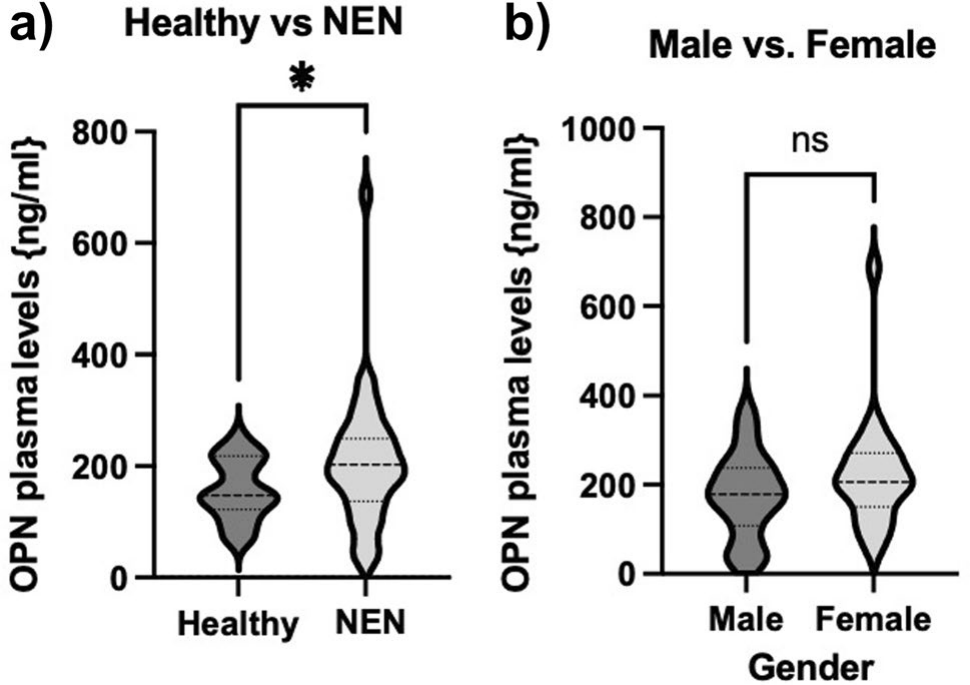
Plasma OPN levels are elevated in Patients with NEN. **a** Plasma concentration of Osteopontin was significantly higher in patients with neuroendocrine malignancies as compared to healthy controls. **b** OPN plasma concentrations revealed no influence of patient gender. Violin plots are shown; the median is indicated by the bold dotted line, the thin dotted lines indicate the quartiles. (Unpaired *t*-test p (*) < 0.05). OPN, osteopontin; NEN, neuroendocrine neoplasms; NEC, neuroendocrine carcinoma)

As mentioned above, several studies have already shown the relevance of OPN as a circulating biomarker in different cancer types. To evaluate, whether OPN has a diagnostic and prognostic significance in patients with neuroendocrine malignancies, we compared plasma levels from patients with healthy controls. We observed that OPN levels were significantly higher in patients with NEN (*n* = 38, range 32.3–687 ng/ml) compared to healthy controls (*n* = 10, range 69.8–225.4 ng/ml) (Fig. 1a). There was no difference in OPN levels between male and female patients (Fig. 1b).

### OPN levels are significantly higher in patients with high-grade NEN

**Fig. 2:**
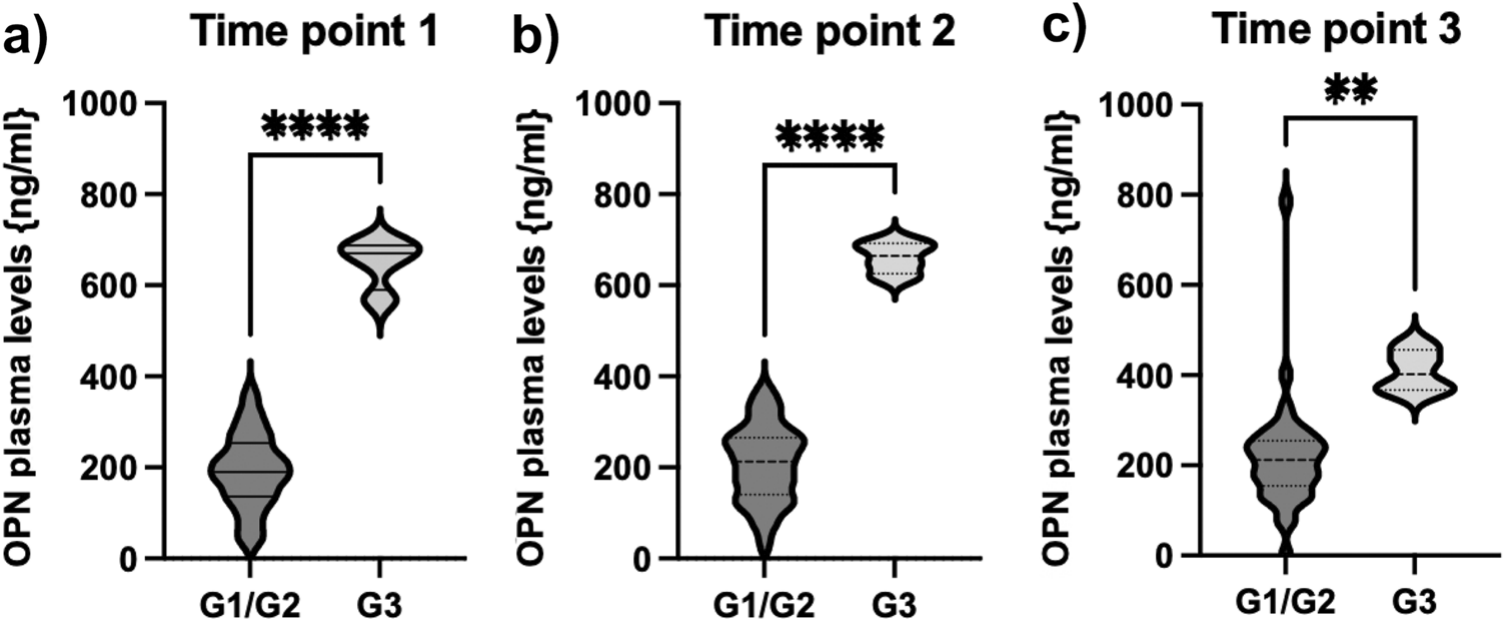
OPN levels are significantly elevated in patients with G3 tumors. **a** The concentration of OPN was significantly higher in patients with G3 tumors throughout the course of disease and therapy in our patient cohort (**a**–**c**). Violin plots are displayed; the median is indicated by the bold dotted line, the thin dotted lines indicate the quartiles. (Unpaired *t*-test *p* (****) < 0.0001; *p* (**) < 0.003)

In order to further analyze the relevance of OPN in neuroendocrine neoplasms, we compared circulating OPN levels in patients with G3 NENs (Ki67 > 20%) with patients diagnosed with well-differentiated G1 and G2 NEN. We found that OPN levels in patients suffering from G3 NEN (range 655–688 ng/ml; median 670.5) were significantly higher as compared to low and intermediate grade tumors (grade 1 and 2, range 32.3–368, median 190.7). This was consistent throughout three different time points of the course of disease and therapy (Fig. 2).

### OPN plasma concentrations show no correlation to primary tumor location

**Fig. 3:**
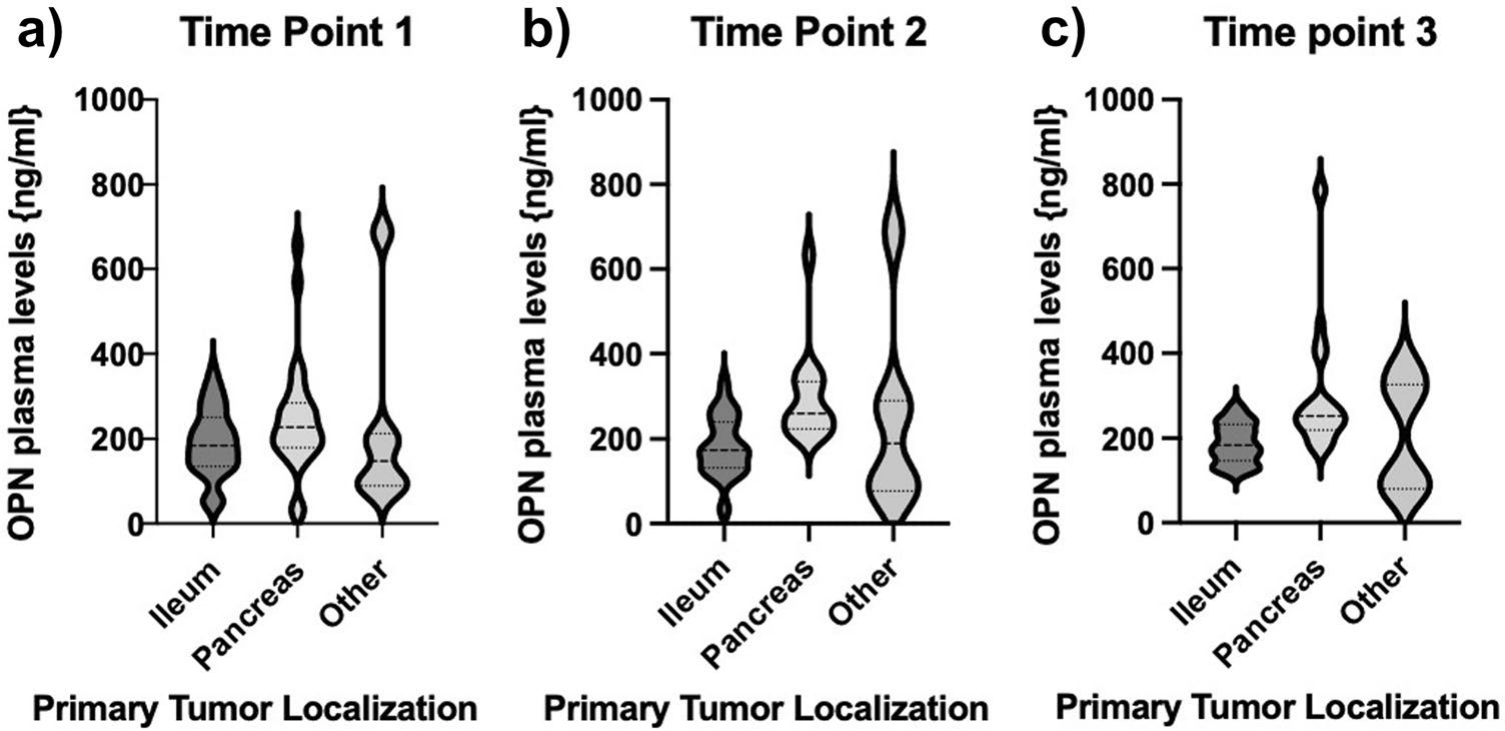
Relevance of primary tumor localization. Circulating concentrations of OPN showed no correlation with primary tumor location. This could be observed at all three different time points when analyses were performed (**a–c**). Violin plots are displayed; the median is indicated by the bold dotted line, the thin dotted lines indicate the quartiles (Unpaired *t*-test, no significance)

Next, we sought to analyze, whether OPN plasma concentrations showed changes in patients with different primary tumor localizations. We analyzed three groups—patients with pancreatic or ileal primary, being the most prevalent entities in our cohort, compared to other localizations. Our analysis revealed that OPN levels were not associated with the localization of the primary tumor, which was consistent at three different time points during the course of disease and therapy (Fig. 3).

### OPN levels do not predict therapeutic response

**Fig. 4:**
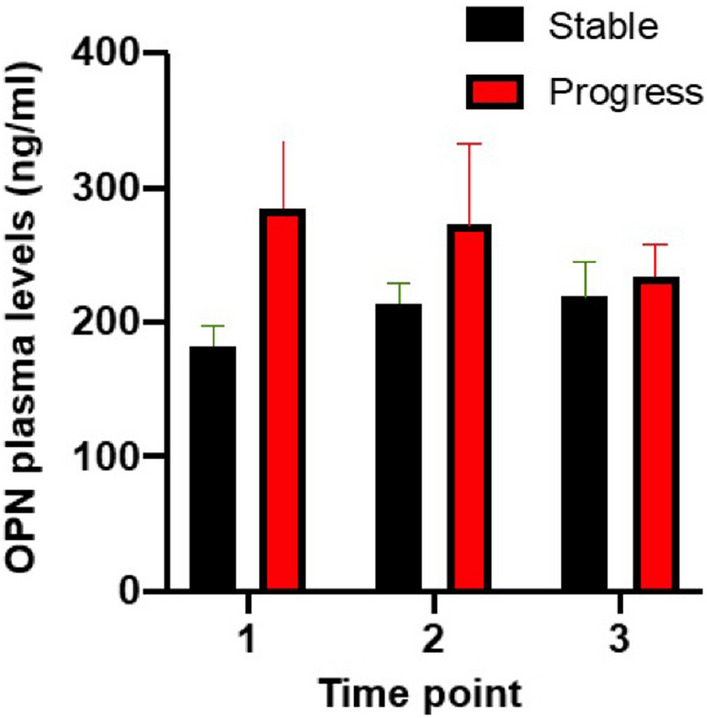
OPN levels and therapeutic response. OPN plasma levels were evaluated at three different time points and patients who remained stable were compared to patients that showed progressive disease. No significant correlation was found

When looking at the longitudinal measurements of OPN levels in individual patients, there was no significant correlation between OPN and treatment response. In most cases, OPN levels remained stable or showed a slight decline throughout the course of the observation time (Fig. 4).

### OPN plasma concentrations in correlation to corresponding Chromogranin A and NSE levels

**Fig. 5:**
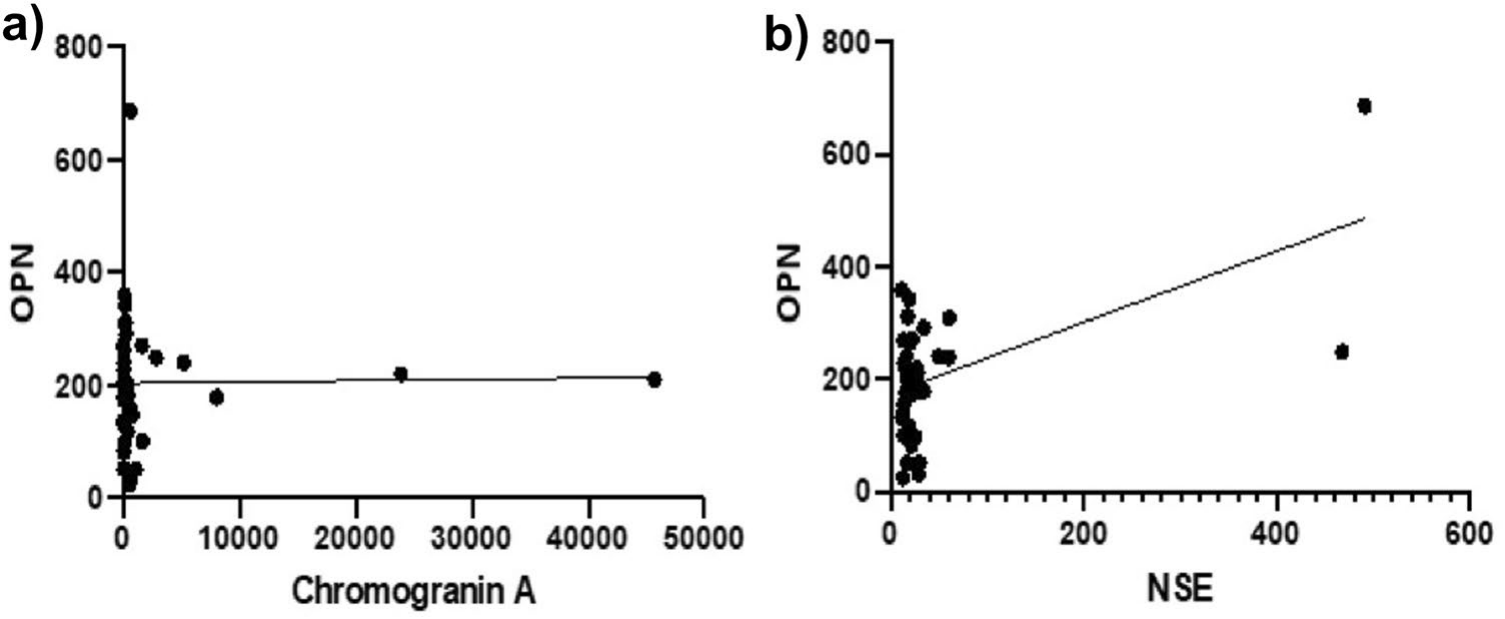
Correlation of OPN plasma levels (ng/ml) with Chromogranin A (CgA; ng/ml) and Neuron specific enolase (NSE; μg/l) levels. Circulating OPN levels were compared to corresponding CgA and NSE values at the start of surveillance (a&b). No correlation was found between OPN and CgA levels. OPN and NSE levels showed a significant correlation (p (***) < 0.0003)

We next tested whether the circulating OPN levels correlated with the current standard biomarker for NEN, Chromogranin A (CgA) or to NSE (Fig. 5). While there is no association between OPN and CgA, we found a significant correlation between the levels of OPN and NSE (*p* < 0.0003; *r* value 0.5668).

### OPN plasma concentrations in correlation to corresponding Ki-67 levels

**Fig. 6:**
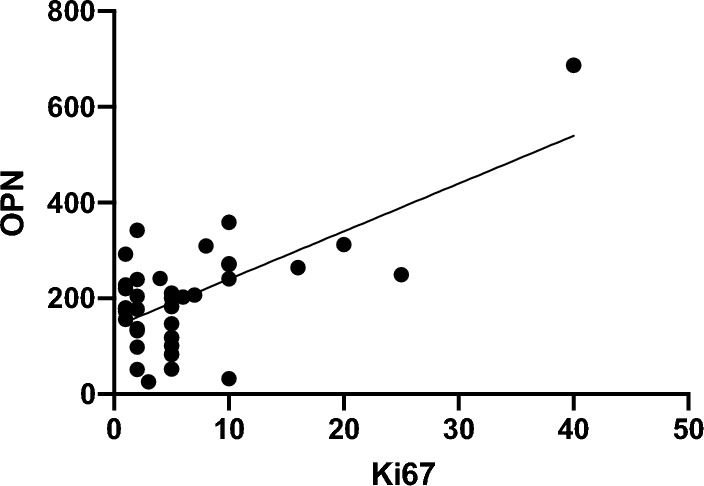
Correlation of OPN plasma levels (ng/ml) with Ki-67 levels (%). Circulating OPN levels were compared to corresponding Ki-67 values at the start of surveillance. We found a significant correlation between OPN and Ki-67 levels

Subsequently, we wanted to find out whether circulating OPN levels correlated with Ki-67 (Fig. 6). We found a significant correlation between the levels of OPN and Ki-67 (*p* < 0.0001; *r* value 0.6769).

### Association of OPN levels with progression-free survival

**Fig. 7:**
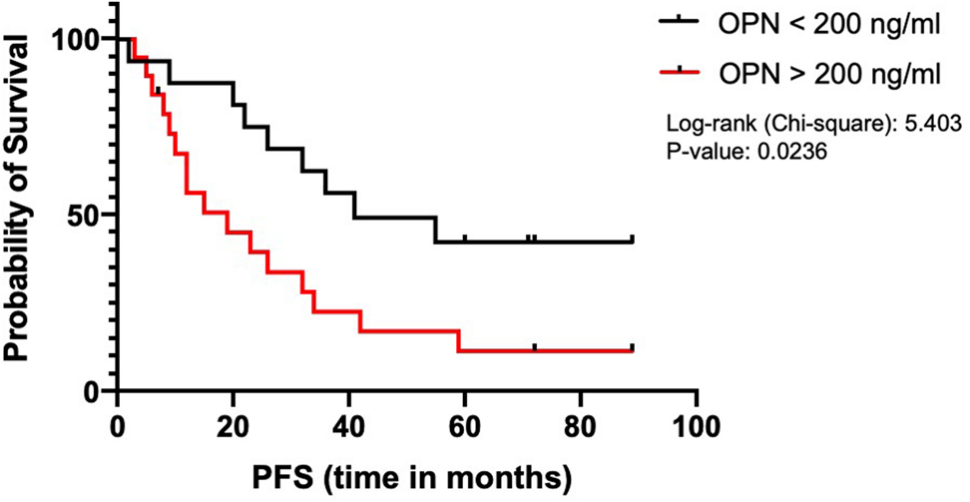
Correlation of circulating OPN levels with progression free survival (PFS). When using a cutoff concentration of 200 ng/ml we observed that lower plasma OPN concentrations indicated significantly longer PFS

Finally, we asked whether circulating plasma OPN levels could indicate enhanced or worsened progression-free survival. Using a cutoff value of 200 ng/ml OPN, we compared the progression-free survival (PFS) in patients that had values below (*n* = 18) versus above (*n* = 20) the cutoff. Using Kaplan–Meier analysis, we found that Osteopontin indicates a significantly worsened prognosis with shorter PFS when using a cutoff value of 200 ng/ml (Fig. 7). The median PFS was 41 months in the group of patients with OPN levels below versus 19 months in individuals with OPN levels above the cutoff value (hazard ratio 0.38; *p* < 0.03).

**Fig. 8:**
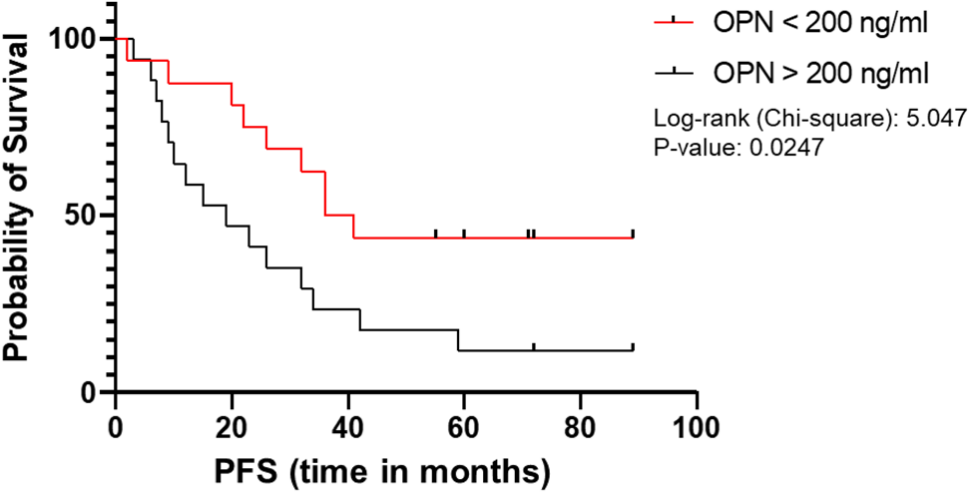
Correlation of circulating OPN levels with Progression free survival (PFS) in G1 and G2 cases. When using a cutoff concentration of 200 ng/ml we observed that lower plasma OSP concentrations still indicated significantly longer PFS

Since we had only a limited number of patients with G3 tumors, we asked whether within the well differentiated G1/G2 cases high OPN correlates with PFS. Indeed, we found that OPN still indicates a significantly worsened prognosis with shorter PFS when using a cutoff value of 200 ng/ml (Fig. 8). The median PFS was 38.5 months in the group of patients with OPN levels below versus 19 months in individuals with OPN levels above the cutoff value (hazard ratio 0.39; *p* < 0.02).

Thus, OPN can be used as an additional parameter to stratify the risk of progression also in patients with well-differentiated G1/G2 tumors.

## Discussion

In the current study, we evaluate OPN as a biomarker for neuroendocrine malignancies and demonstrate that a plasma OPN level above 200 ng/ml is associated with a significantly shorter PFS in patients with NEN. High OPN levels obtained at the initial analysis indicate a more aggressive tumor biology and predict the adverse disease outcome.

OPN is a protein, which is mainly synthesized by osteocytes, osteoblasts and hematopoietic cells (Zhao et al. 2018). It has been linked to inflammatory processes, and it has been previously shown that high OPN levels can estimate severity of disease and risk of mortality in critically ill patients (Roderburg et al. 2015).

The relevance of OPN as a marker of cancer aggressiveness has been reported in several malignancies, including breast, colorectal, pancreatic, lung, bladder, oral, head and neck cancer, and several other cancer types (Weber et al. 2010; Wisniewski et al. 2019; Petrik et al. 2006; Loosen et al. 2019).

Neuroendocrine malignancies are a group of rare, very heterogeneous tumors (Detjen et al. 2021). Whereas patients with well-differentiated tumors (G1-G3) show a rather good prognosis, survival in patients with less differentiated tumors (NEC) is poor in most cases (Milione et al. 2017).

Currently, Chromogranin A is established as a circulating biomarker for Neuroendocrine Neoplasms. While Chromogranin A is widely used to monitor the course of disease and response to therapy in individual patients, the absolute levels do not correlate with the prognosis. Indeed, more aggressive tumors can lose CgA production (Kidd et al. 2016). Therefore, new predictive biomarkers are urgently warranted for neuroendocrine malignancies.

To address this issue, we analyzed levels of OPN in plasma from patients with Neuroendocrine Neoplasms at three different time points during the course of disease and therapy. We were able to demonstrate that OPN levels are significantly higher in patients with NEN as compared to healthy controls. Further, OPN levels in patients with high-grade neuroendocrine carcinomas (Grade 3) were significantly higher as compared to low and intermediate-grade tumors (Grade 1 and 2). There was no difference in OPN levels between male and female patients, and we found no correlation to primary tumor localization. Importantly, while the levels correlated with the prognosis, they did not show a correlation with the tumor burden or response to therapy. Thus, OPN levels appear to reflect the tumor biology in terms of the aggressiveness of the tumor cells, irrespective of the initial tumor size.

When correlating OPN levels in plasma obtained at the first consultation with corresponding CgA and NSE levels, we observed a significant correlation to NSE values, while no correlation with CgA could be found. This verifies existing data concerning the relevance of elevated NSE levels in reflecting a worsened disease course (van Adrichem et al. 2016).

CgA is highly expressed in well-differentiated NEN and therefore the concentration does not necessarily reflect the aggressiveness of the tumor. Moreover, CgA levels can be affected by confounders such as renal insufficiency, atrophic gastritis, and during therapy with proton pump inhibitors (Mettler et al. 2022).

We therefore propose that OPN provides additional valuable information about the tumor biology of NEN, with aggressive tumors correlating with higher OPN levels.

We also sought to find out, whether OPN levels were correlated to Ki-67 values. We found a significant correlation. This may be a key advantage in cases where tumor tissue is not easily accessible for a biopsy or after several lines of tumor therapy.

In the current study, an important aspect is the demonstrated prognostic value of OPN for progression-free survival when using a cutoff value of 200 ng/ml.

Similar results were found in a meta-analysis examining the relevance of OPN for the prediction of overall survival in gastric cancer: in cases with high expression levels of OPN, there was a correlation with factors that mirror more aggressive and advanced disease (i.e., TNM stage, lymph node and distant metastases) (Gu et al. 2016). Our data are also in line with another previous study showing that OPN levels are significantly elevated in patients with metastasized colorectal cancer when compared to healthy controls (Loosen et al. 2018). Further, high pre- and postoperative plasma levels of OPN reveal worse prognosis following tumor resection (Loosen et al. 2018). Together these data suggest that OPN may be used as a prognostic pan-cancer marker, which also includes rare tumor entities such as NEN.

In addition to its role as a biomarker, the mechanistic role of OPN during tumor progression has been suggested. Interestingly, in a study by Ishigamori et al. examining OPN knockout mice with *APC* deficiency, tumor development was shown to be significantly suppressed, whereas in solely *APC*-deficient mice the expression of OPN was upregulated in colon cancers (Ishigamori et al. 2017). Similarly, ablation of OPN in mice infected with *H. pylori* led to a significant decrease of the development of gastric cancer compared to wild-type mice (Lee et al. 2015). Further, in another study analyzing the incidence of chemically induced hepatocellular cancer, OPN deficiency lead to a significant reduction. This seems to be caused by suppression of EGFR-mediated anti-apoptotic signaling (Lee et al. 2016). Together these data indicate that OPN affects cellular proliferation and survival.

Our data here reveal that NEN with high proliferative activity show particularly high OPN levels. It will be interesting to investigate whether OPN signaling promotes NEN cell proliferation and thus contributes to the high proliferative activity, or whether the highly proliferative tumors promote immunological responses that are linked to high OPN values.

In clinical patient care routine, diagnosis and evaluation of cancer largely depends on clinical and histological criteria. Nevertheless, it is important to keep in mind, that blood biomarkers are easily obtainable and may provide additional information. Our data suggest that OPN may serve as a surrogate biomarker for a tissue biopsy if sufficient material cannot be collected, for example, when tissue is not easily obtainable due to high risk of adverse events.

Further, we postulate, that elevated OPN levels at the time of diagnosis of Neuroendocrine Neoplasms can aid with the decision towards a more powerful treatment regimen than are necessary for patients with low OPN levels.

A limitation of our study is the rather small patient cohort. Thus, in the next step, the relevance of OPN should be tested in a larger study group for validation of our results, and to evaluate, whether the combinational analysis of OPN with currently used markers can be used to identify high-risk patients. Hence this may influence the diagnostic and therapeutic workflows and impact the outcome of patients with NEN.

## Conclusion

Our data demonstrate for the first time that circulating OPN may be considered as a prognostic biomarker in patients with neuroendocrine malignancies in order to identify patients with potentially lower progression free survival. This biomarker is easily obtainable non-invasively at any time point and may help in guiding treatment decisions in the future. However, further investigation including larger cohorts of NET and NEC patients are necessary in order to fully understand the pathophysiological role of OPN in this cancer type before implementation into clinical algorithms can be considered.

## Data Availability

The data presented in the current study are available from the corresponding author on reasonable request.
